# Supplementary material for: The typhoid Mary legacy: Genomic epidemiology uncovers contemporary carriage dynamics across two decades of enteric fever surveillance in England and Wales
Source: PLoS Negl Trop Dis. 2026 Apr 27;20(4):e0014177. doi: 10.1371/journal.pntd.0014177 (PMC13132454; doi:10.1371/journal.pntd.0014177)
Supplement: S1 Table — (PDF) [file pntd.0014177.s001.pdf]

S1 Table. Overview of variables and missing data included in statistical analysis.

Variable	Recorded Values	Missing/Unknown Values (%)
Carriage Status	8297	0 (0.0)
Patient Sex	8200	97 (1.2)
Patient Age Group	8258	39 (0.5)
Travel Occurrence	7743	554 (6.7)
Vaccination Status	4012	4285 (51.6)
Antibiotic Therapy	5862	2435 (29.3)
Causative Serovar	8297	0 (0.0)
Month of Sampling	8297	0 (0.0)
