# Supplementary material for: The typhoid Mary legacy: Genomic epidemiology uncovers contemporary carriage dynamics across two decades of enteric fever surveillance in England and Wales
Source: PLoS Negl Trop Dis. 2026 Apr 27;20(4):e0014177. doi: 10.1371/journal.pntd.0014177 (PMC13132454; doi:10.1371/journal.pntd.0014177)
Supplement: S2 Table — (PDF) [file pntd.0014177.s002.pdf]

S2 Table. Raw results from logistical regression model for statistical analysis of factors contributing to carriage of typhoidal *Salmonella*.

Variable	Categories	Odds Ratio	Std. Err.	t	P>|t|	[95% Conf. Interval]	
Cat_Sex		0.94	0.13	-0.46	0.648	0.72	1.23
Cat_Age (Ref: 0-10)							
	11–20	0.95	0.20	-0.25	0.804	0.62	1.45
	21–30	0.65	0.14	-2.00	0.046	0.43	0.99
	31–40	0.85	0.19	-0.72	0.471	0.56	1.31
	41–50	0.87	0.23	-0.53	0.598	0.52	1.46
	51–60	0.93	0.29	-0.24	0.808	0.51	1.70
	61–70	0.65	0.31	-0.89	0.373	0.26	1.66
	71–80	1.10	0.60	0.17	0.864	0.38	3.18
	81–90	5.47	2.57	3.62	0.000	2.18	13.72
	91+	4.46e-06	0.00	-0.02	0.985	0	0
Cat_Travel		0.61	0.14	-2.24	0.025	0.39	0.94
Cat_Vacc		1.11	0.25	0.45	0.656	0.71	1.73
Cat_Treatment		0.71	0.26	-0.93	0.355	0.35	1.46
Cat_Serovar (Ref: Typhi)							
	Paratyphi A	0.65	0.11	-2.58	0.010	0.47	0.90
	Paratyphi B	1.37	0.40	1.08	0.282	0.77	2.41

All results adjusted also for restricted cubic spline
