# Supplementary material for: The typhoid Mary legacy: Genomic epidemiology uncovers contemporary carriage dynamics across two decades of enteric fever surveillance in England and Wales
Source: PLoS Negl Trop Dis. 2026 Apr 27;20(4):e0014177. doi: 10.1371/journal.pntd.0014177 (PMC13132454; doi:10.1371/journal.pntd.0014177)
Supplement: Attachment — Submitted filename: PLos one typoid Mary review.docx [file pntd.0014177.s011.docx]

**Nisbet et al.,** (Manuscript Number: PNTD-D-25-01911)

To study the carriage of typhoidal Salmonella, inclusive of Salmonella Typhi and Paratyphi A/B, Nisbet et al., collected data from the United Kingdom Health Security Agency’s Salmonella reference laboratory of 8,297 cases of enteric fever between 2004-2023 and analyzed the data extensively to track disease trends and identify carriers. Authors studied prevalence of disease by three different causative agents of enteric fever and identified number of Carriage Infections Cases progressing to carriage. Overwhelming majority (92%) of cases reported travel, mainly to South Asia where disease is most prevalent in more deprived communities. The older population between 81 and 90 years of age had significant higher odds of carriage and even if they did not travel within 28 days of disease onset. The odds were significantly lower in patients aged 21-30 and in S. Paratyphi A infections.

Among antibiotics used for enteric fever, cephalosporins were the most prescribed antibiotic for acute and carriage infections. No significant difference was observed in antibiotics prescribed for acute and carriage infections.

This study highlights increased prevalence of acute enteric fever infection in more deprived communities, and elevated risk of TS carriage in elderly patients and those without recent foreign travel. The host immune systems seem to play a role in disease carriage versus the genotype of bacterial infection.

This is an excellent study with wide implication in understanding and managing typhoid disease spread. I don’t have specific comment and recommend publication in Plos one.
