# Supplementary material for: The typhoid Mary legacy: Genomic epidemiology uncovers contemporary carriage dynamics across two decades of enteric fever surveillance in England and Wales
Source: PLoS Negl Trop Dis. 2026 Apr 27;20(4):e0014177. doi: 10.1371/journal.pntd.0014177 (PMC13132454; doi:10.1371/journal.pntd.0014177)
Supplement: Attachment — Submitted filename: PNTD-D-25-01911 manuscript review 02.10.2026.docx [file pntd.0014177.s012.docx]

**Manuscript review:**

**The typhoid Mary legacy: genomic epidemiology uncovers contemporary carriage dynamics across two decades of enteric fever surveillance in England and Wales**

**Manuscript summary:**

This manuscript examines enteric fever surveillance data from England and Wales spanning 2004-2023, with a focus on understanding bacterial carriage dynamics of typhoidal Salmonella (S. Typhi and S. Paratyphi A/B). The study analyzed 8,297 cases and identified that approximately 2.7% of infections progressed to carriage (persistence >3 weeks), with only 0.1% becoming chronic carriers (>12 months). Key findings revealed that carriage risk was significantly elevated in elderly patients (ages 81-90) and those without recent foreign travel, while patients aged 21-30 and those infected with S. Paratyphi A had reduced carriage odds. Importantly, phylogenomic analysis found no distinct genetic signatures associated with carriage isolates, suggesting that host immune factors rather than bacterial genotype play a more critical role in determining whether infections persist long-term.

**General Comments:**

The manuscript is organized and written well, with no need for extensive language edits.

The study acknowledges substantial missing data across multiple variables (referenced in S1 Table), which required multiple imputation for statistical analysis. While I believe the authors used appropriate statistical methods to address this, missing data can still introduce bias and reduce the analytical power of the study overall.

**Manuscript Limitations:**

Figure 3:

The 28-day travel window prior to disease onset may not capture all travel-associated cases, as the authors note that extending this to 60 days previously identified an additional 12% of cases as travel-related. This could affect the accuracy of travel versus non-travel associations with carriage.

What was the rationale for limiting the travel window to 28 days?

Figure 5:

Despite concluding that host immune factors likely play a greater role than bacterial genotype in carriage, the study lacks direct examination of patient immune status, comorbidities, or other host characteristics beyond age. The hypothesis about immune function is inferred rather than directly tested.

Are any data available that can be included (such as comorbidities, general immune status) to begin to infer additional host factors, other than age?

Figure 7:

Only 46% of S. Typhi cases, 36% of S. Paratyphi A cases, and 55% of S. Paratyphi B cases had publicly available high-quality genomes for phylogenetic analysis. This incomplete coverage could potentially miss important genetic patterns that may be associated with trends in carriage among the cases studied.

Are further studies planned to analyze these data as more genomes become publicly available? I believe this future re-analysis, combined with any additional subject data (underlying health conditions, etc. as mentioned above) will provide interesting additional data regarding carriage trends.

Conclusion:

While there are clear limitations to this study, as mentioned above, this analysis spanning 20 years of data in England and Wales captures a robust set of data examining long term trends, seasonal changes and the overall temporal stability of this disease (with post-pandemic incidence increase noted). Overall, this study highlights a few key public health findings, relating to socioeconomic impacts on the spread of this disease, as well as the higher disease burden in more deprived communities – informing where more intervention through vaccination may be needed. Importantly, the authors openly discuss the challenges and limitations of the data and do not overextend their conclusions.
