# Supplementary material for: The typhoid Mary legacy: Genomic epidemiology uncovers contemporary carriage dynamics across two decades of enteric fever surveillance in England and Wales
Source: PLoS Negl Trop Dis. 2026 Apr 27;20(4):e0014177. doi: 10.1371/journal.pntd.0014177 (PMC13132454; doi:10.1371/journal.pntd.0014177)
Supplement: Attachment — Submitted filename: Response to Reviewers 17.03.26.docx [file pntd.0014177.s014.docx]

Dear Sanjai Kumar

Thank you for your consideration of our manuscript and for the reviewers’ thoughtful and constructive feedback. We appreciate their recognition of the study’s strengths, including the merit of our exceptional surveillance dataset and the novelty of key findings that represent a substantial contribution to the field.

In response to the reviewers’ comments, we are pleased to say that revisions have been made to the manuscript, and we present our point-by-point response to address each comment below. Please note that all line numbers below refer to those in the track-changes version of manuscript.

**Reviewer 1**

**1. “Provide better justification for the 3-week threshold and discuss limitations”**

The 21-day threshold for distinguishing acute and carriage infections is not based on pathophysiology but on UK-specific practical public health management grounds, based on the incubation period of typhoidal *Salmonella*, and usual period of infection. It is used here to ensure that carriers can be identified during the very early stages of infection and monitored accordingly. This method of defining carriage has been widely cited by authors including Chattaway [5] and Gunn [8]. These references have been reiterated where the carriage definitions are cited on lines 568-569.

**2. “Authors needs to address methodological limitations around carriage definition and potential surveillance biases”**

Identification of carriage utilising repeat isolates is limited, however repeat isolation remains the most feasible approach for identification within surveillance systems. The manuscript has been updated to discuss this (lines 396-404) and now reads: “The data presented here may underestimate TS carriage due to reliance on repeated isolates from patients. Identification requires individuals to present to healthcare services on which introduces potential surveillance bias, with more socially disadvantaged groups tending to present later in the course of illness [32], and as presentation is often driven by symptoms, asymptomatic carriers are likely to be missed. Furthermore, the intermittent nature shedding during carriage means organisms may not be detected within samples from a carrier individual. Nevertheless, repeat isolation remains the most feasible approach for identification within surveillance systems.”

**3. “Include more discussion of immunological factors that might explain age-related carriage patterns”**

We agree that immunological factors likely have an affect on typhoidal *Salmonella* carriage and have discussed this on lines 407-413 which reads: “Children aged 0-10 were instead identified as the most frequent carrier patients, alongside significantly increased odds of carriage in those aged 81-90 – groups likely possessing reduced immune capacity due to naivety and immunosenescence respectively [37-39]. Given those aged 21-30, likely possessing resilient immune systems, show significantly decreased odds of TS carriage, we hypothesise that the immune capacity of a patient is likely a key driving factor regarding whether an infection can be cleared or persists long term.” The literature regarding immunological factors affecting carriage is very limited and given that no information regarding patient immune system was available within this study more detailed discussion on the matter is outside the scope of the project.

**4. “Fig 1 showed data for 2006-2023. Is there any reason 2004-2005 data were excluded?”**

Figures regarding antibiotic utilisation, and vaccination status, was not available from cases prior to 2006, as this data was not routinely collected. This has been clarified within the manuscript (lines 541-543) which reads: “Metadata regarding *S.* Typhi vaccination status and antibiotic regimes taken for enteric fever treatment were available from 2006.”. These variables were included in our statistical model alongside the restricted cubic spline capturing temporal trends over time, hence should not majorly affect the overall results. To confirm this, we utilised 4 datasets, including both the previously presented spline and a reduced spline to minimise extreme trend estimated:

1. The full dataset alongside a spline with knots at 12, 39, 66, 93, 120, 147, 174, 201, 228 months (as presented in the original submission)
2. The full dataset alongside a spline with knots at 12, 93 and 74 months
3. The dataset without antibiotic utilisation and vaccination status variables alongside a spline with knots at 12, 39, 66, 93, 120, 147, 174
4. The dataset without antibiotic utilisation and vaccination status variables with a spline with knots at 12, 93 and 74 months

Missing data from each was imputed and analysed as previously described. The resulting odds ratios did not change substantially (<10% - Odds ratios can be seen below), indicating no meaningful difference. Hence, we propose the model is robust for all variables and therefore the full dataset—with the knots at 12, 93 and 74 months which best represent the average trend over time - will be retained within the publication. The methodology within the manuscript has been updated to reflect this (lines 590-593) and now reads “The model also included a restricted cubic spline, based on the sample month (1-240) for each case’s representative isolate, to assess non-linear trends over the 20 year-period, with knots at 12, 93 and 174 months. “, alongside updates to reported statistical values throughout the manuscript.

| Variable | Categories | 1 | 2 | 3 | 4 |
| --- | --- | --- | --- | --- | --- |
| Sex |  | 0.94 | 0.94 | 0.94 | 0.94 |
| Age | 11-20 | 0.95 | 0.95 | 0.94 | 0.94 |
|  | 21-30 | 0.65 | 0.65 | 0.65 | 0.65 |
|  | 31-40 | 0.86 | 0.85 | 0.84 | 0.84 |
|  | 41-50 | 0.86 | 0.87 | 0.85 | 0.86 |
|  | 51-60 | 0.93 | 0.93 | 0.93 | 0.93 |
|  | 61-70 | 0.65 | 0.65 | 0.65 | 0.65 |
|  | 71-80 | 1.11 | 1.10 | 1.14 | 1.14 |
|  | 81-90 | 5.36 | 5.47 | 5.72 | 5.76 |
|  | 91+ | 4.42e-06 | 4.46e-06 | 3.46e-06 | 9.93e-06 |
| Travel |  | 0.60 | 0.61 | 0.60 | 0.60 |
| Vaccination |  | 1.14 | 1.11 | N/A | N/A |
| Antibiotic |  | 0.71 | 0.71 | N/A | N/A |
| Serovar | Paratyphi A | 0.65 | 0.65 | 0.67 | 0.67 |
|  | Paratyphi B | 1.33 | 1.37 | 1.45 | 1.47 |

**“Please check typographical errors, like in Line 505: "Welsh"”**

The spelling “Welsh” is correct in the context (line 543) “The English and Welsh indices of multiple deprivation 2019 [35, 36] determined patients’ IMD decile based on their home postcode: IMD decile 1 and IMD decile 10 corresponding to the most and least deprived areas, respectively.” as it refers to the population of Wales with regards to the Welsh indices of deprivation. ([Welsh Index of Multiple Deprivation 2025 | GOV.WALES](https://www.gov.wales/welsh-index-multiple-deprivation-2025-series)). Other typological errors have been corrected where identified.

**Reviewer 3**

**“ What was the rationale for limiting the travel window to 28 days?”**

The 28 day timeframe originates from the UKHSA public health operational guidelines, which base this period on national enhanced surveillance data showing 95% of cases with a travel history had symptom onset within this period ([Public health operational guidelines for typhoid and paratyphoid (enteric fever) - GOV.UK](https://www.gov.uk/government/publications/typhoid-and-paratyphoid-public-health-operational-guidelines/public-health-operational-guidelines-for-typhoid-and-paratyphoid-enteric-fever)). The exact definition of travel associated has been clarified within the manuscript (line 535-541) which now reads: “Travel associated cases were determined in line with UKHSA operational guidelines [59]. Under these, cases with symptom onset prior to return from or within 28 days of foreign travel to an endemic region are considered travel associated. Cases with onset 28-60 days post travel to an endemic region may also be assessed to determine whether travel was relevant to the infection. Cases with travel reported >60 days before onset, or to non-endemic regions, may also be classified as travel associated based on specific case details, in line with public health guidelines.”.

Additional data has been obtained to indicate the exact percentage of patients reporting symptoms prior to or within 28 days of return/arrival and these findings have been added to the manuscript at line 191-193 which now reads: “Where cases provided a UK return/arrival date, 96% (5333/5552) reported symptom onset prior to or within 28 days of return/arrival.” and at line 245-247 which now reads: “Where a UK return/arrival date was specified, 95% (142/150) of carriage cases reported symptom onset prior to return or within 28 days of travel, similar to the 96% (5191/5402) in acute cases.”. Minor changes to wording describing travel association have also been made throughout the document in line with this, and within the discussion on line 437-444 which now reads “Previous UKHSA investigations have noted that 12% of cases initially classified as non-travel associated can be linked to foreign travel when the assessment period was extended to >60 days [7]. This suggests that some individuals may have been infected abroad, and remained asymptomatic upon their return, before developing symptoms later, leading to delayed disease reporting. As case details are not always available beyond 60 days, relevant travel information may not be captured, contributing to lower reported travel in carriers.”

**“Are any data available that can be included (such as comorbidities, general immune status) to begin to infer additional host factors, other than age?”**

Data regarding patient immune status or underlying health conditions is not routinely collected by UKHSA, so further analysis on host factors is outside the scope of this study.

**“ Are further studies planned to analyze these data as more genomes become publicly available? I believe this future re-analysis, combined with any additional subject data (underlying health conditions, etc. as mentioned above) will provide interesting additional data regarding carriage trends”**

A statement regarding this missing data has been added into the manuscript at lines 267-270, which now reads: “Assembly availability was limited across all serovars due to routine UKHSA WGS only commencing in April 2014 [16], and the need for Enterobase assemblies to meet previously described quality control metrics for publication[19].” We aim to explore the addition of more sequencing data, and metadata in future projects.

**Note:**

In line with journal requirement requests, the base map for Figures 3,6 and S4 has been specified and a detailed description of their generation included to clarify the lack of copyright issues - alongside adjustments to the financial disclosure and competing interests’ statements. These are maintained as tracked changes within the tracked changes manuscript.

**References**

[5] Chattaway MA, Gentle A, Nair S, Tingley L, Day M, Mohamed I, *et al*. Phylogenomics and antimicrobial resistance of *Salmonella* Typhi and Paratyphi A, B and C in England, 2016-2019. Microbial Genomics. 2021; 7(8): 1-15. doi: 10.1099/mgen.0.000633.

[7] Vusirikala A, Katwa P, Kirkbride H, Dabke G, Taye A, Krisifoe S, *et al*. Epidemiology of non-travel enteric fever cases in England: insights from 10 years of surveillance. Public Health. 2024; 234: 43-6. doi: 10.1016/j.puhe.2024.05.035.

[8] Gunn JS, Marshall JM, Baker S, Dongol S, Charles RC, Ryan ET. Chronic carriage: epidemiology, diagnosis, and gallbladder persistence. Trends in Microbiology. 2014; 22(11): 648-55. doi: 10.1016/j.tim.2014.06.007.

[16] Chattaway MA, Dallman TJ, Larkin L, Nair S, McCormick J, Mikhail A, *et al*. The Transformation of Reference Microbiology Methods and Surveillance for *Salmonella* With the Use of Whole Genome Sequencing in England and Wales. Frontiers in Public Health. 2019; 7: 317. doi: 10.3389/fpubh.2019.00317.

[19] Dyer NP, Pauker B, Baxter L, Gupta A, Bunk B, Overmann J, *et al*. EnteroBase in 2025: exploring the genomic epidemiology of bacterial pathogens. Nucleic Acids Res. 2025; 53(D1) :D757–D62. doi: 10.1093/nar/gkae902.

[32] Cookson R, Propper C, Asaria M, Raine R. Socio-Economic Inequalities in Health Care in England. Fiscal Studies. 2016; 37(3-4): 371-403. doi: 10.1111/j.1475-5890.2016.12109.

[35] Government of the United Kingdom. English indices of deprivation 2019 [Internet]. London: Government of the United Kingdom; 2019 Sep 26 [cited 2025 Oct 10]. Available from: https://www.gov.uk/government/statistics/english-indices-of-deprivation-2019.

[36] Welsh Government. Welsh Index of Multiple Deprivation (full Index update with ranks): 2019 [Internet]. Cardiff: Welsh Government; 2020 Oct 1 [cited 2025 Oct 10]. Available from: https://www.gov.wales/welsh-index-multiple-deprivation-full-index-update-ranks-2019.

[37] Simon AK, Hollander GA, McMichael A. Evolution of the immune system in humans from infancy to old age. Proceedings of the Royal Society B: Biological Sciences. 2015; 282(1821): 20143085. doi: 10.1098/rspb.2014.3085.

[38] Wrona MV, Ghosh R, Coll K, Chun C, Yousefzadeh MJ. The 3 I's of immunity and aging: immunosenescence, inflammaging, and immune resilience. Frontiers in Aging. 2024; 5: 1490302. doi: 10.3389/fragi.2024.1490302.

[39] Weyand CM, Goronzy JJ. Aging of the Immune System. Mechanisms and Therapeutic Targets. Annals of the American Thoracic Society. 2016; 13 (Suppl 5): S422-S8. doi: 10.1513/AnnalsATS.201602-095AW.

[59] United Kingdom Health Security Agency.Public health operational guidelines for typhoid and paratyphoid (enteric fever) [Internet]. London: United Kingdom Health Security Agency; 2024 Jan 3 [cited 2025 Oct 10]. Available from: https://www.gov.uk/government/publications/typhoid-and-paratyphoid-public-health-operational-guidelines/public-health-operational-guidelines-for-typhoid-and-paratyphoid-enteric-fever.
